# Financial satisfaction, food security, and shared meals are foundations of happiness among older persons in Thailand

**DOI:** 10.1186/s12877-023-04411-1

**Published:** 2023-10-24

**Authors:** Sirinya Phulkerd, Rossarin Soottipong Gray, Aphichat Chamratrithirong, Umaporn Pattaravanich, Sasinee Thapsuwan

**Affiliations:** Institute for Population and Social Research, Mahidol University, Nakhon Pathom, Thailand

**Keywords:** Ageing, Financial satisfaction, Food security, Family meals, Older persons, Happiness, Wellbeing

## Abstract

The objective of this study was to investigate the association between happiness and food-related behaviours and other potential factors among older persons in Thailand. This was a cross-sectional study with multistage random sampling. In total, 1,197 older (age 60 + years) persons participated in this study. Face-to-face interviews were conducted to obtain information about socio-demographic characteristics, health status, household chores, home gardening, financial situation, family meals, and food security. Descriptive and multivariate regression analyses were performed for data analysis. The analysis found that age, health status, household chores, and home gardening were found to be significantly associated with happiness. Statistically-significant associations of financial situation, family meals, and food insecurity with happiness were also observed. People who were not dissatisfied with their financial situation were happier than people who were unhappy with their financial situation. Older people who ate every meal with a family member(s) were happiest. People who had severe food insecurity were less happy than those with food security. The findings suggest the need for investment priorities on food and nutrition with a long-term policy to ensure financial self-sufficiency and food security among older persons and their family.

**Supplementary Information:**

The online version contains supplementary material available at 10.1186/s12877-023-04411-1.

## Introduction

Rapidly changing population age structure is being observed worldwide. The increase in the global number of persons age 65 years or older is accelerating, totalling 727 million persons in 2020. That number is expected to exceed 1.5 billion by 2050 [1]. The share of the 65 + years age group of the total is expected to almost double: From 9.3% to 2020 to 16.0% in 2050. This poses a challenge to policy makers and other stakeholders on the ability of countries to ensure of the health and well-being of older persons for as long as possible to prevent a demographic calamity.

Thailand is among seven ASEAN-member countries that have already become an ‘*aged society*,’ and it ranked second highest by proportion of the population age 60 years or older (19% in 2021) following Singapore (22%) [2]. By 2040, it is projected that 1 in 3 Thais will be ‘*older’* persons (i.e., age 60 + years). The share of the population who are older persons is projected to increase from 12.5 million in 2021 to 20.5 million in 2040. Furthermore, the number of persons age 80 years or older is expected to more than double between 2021 and 2040, reaching 3.4 million. As people age, they are more likely to experience various adverse conditions, especially a gradual decrease in physical functioning and mental acuity, and a growing prevalence of chronic disease [3]. If older Thais can live their ‘*sunset years’* in good health, and are able to perform activities in daily living (ADL) without assistance from others, then that should have positive benefits for them, their families, and society at large.

Psychological wellbeing and health in an ageing population are closely related. A growing body of research indicates that the protective influence of an optimistic disposition can lead to positive ageing, such as reducing ill-health, cognitive impairment, and cardiovascular disease, and enable the pursuit of healthy and active lifestyles [4–6]. Positive psychological well-being (e.g., happiness) has a favourable effect on lowering future mortality and morbidity risks in both healthy and diseased populations [7]. Conversely, reductions in happiness can increase the risk of various chronic conditions such as stroke, lung disease, and rheumatoid arthritis in older persons [8]. Accordingly, the concept of happiness in older age is attracting increased attention of researchers and health policy makers.

The World Health Organization (WHO) defines active ageing as ‘*the process of optimizing opportunities for health, participation, and security in order to enhance quality of life as people age* [9].’ Promoting active ageing requires support for the three pillars of well-being: Health, Participation, and Security. The *Health* pillar focuses on reduction of risk factors for chronic disease and decline in physical and mental function, as well as promotion of protective factors as part of a healthy lifestyle. The *Participation* pillar focuses on older people’s participation in society in both paid and unpaid activities such as employment, cultural events, and household activities according to basic human rights, capacities, needs, and preferences. The *Security* pillar focuses on addressing social, financial, and physical security needs, including family care and community care to support ADL. Accordingly, this study analysed the influence of the pillars of active ageing in shaping how individuals and populations live, and how that understanding can inform policy decision making.

Positive psychological well-being is affected by many factors other than health. For example, one of the key determinants of maintaining good health throughout life is food behaviour. Previous studies have found that fruit and vegetable (FV) consumption has beneficial psychological effects [10], and people who had sufficient FV consumption also had higher happiness scores than people who had insufficient FV consumption [11]. Household gardening, especially when cultivating vegetables (i.e., kitchen garden), is also linked to emotional well-being (or happiness) of people across race and place of residence [12]. A kitchen garden can contribute to people’s food security and their livelihoods and liveability. Another dimension of food behaviour is eating meals with family or household members. There is a positive correlation between sharing the family meal at the same time of day and health and well-being outcomes [13]. However, greater urbanization, addiction to social media, and faster-pace lifestyles are eroding the ability of families -- or even couples -- to eat meals together [13, 14].

Financial status is also an important determinant of happiness. Studies have found a positive correlation between lack of adequate income and depression, and the association becomes stronger at the lower-income quintiles [15]. Similarly, another study found a negative association between economic status and happiness, and this was more severe among rural populations [16]. The link between food, economic, and socio-demographic factors becomes more important in older age, if only because the inevitable progressive deterioration of physiological function aggravates any shortcomings in daily life.

More and more analysts are cognizant of the approaching ‘*demographic tsunami’* that is on the horizon and nearing fast. In the case of Thailand, the society may become ‘*completely aged’* before it becomes sufficiently prosperous [2]. Of particular concern is how to cope with the enormous burden of chronic illness/conditions, functional dependence for ADL, and financial insecurity of a rapidly ageing Thai society. Government policies need to address these challenges, while identifying opportunities for older persons to experience healthy or happy ageing whereby they can continue to work in gainful employment and access certain benefits and protections.

According to a 2017 survey by the National Statistical Office (NSO), more than 70% of Thai older persons self-rated their happiness at a ‘moderate’ or ‘lower’ level [17]. It is well known that health and participation in society are associated with happiness of older persons. However, other conditions that can maintain health throughout life, particularly food-related behaviours remain poorly understood. As yet, no studies have identified the food determinants and other potential factors that affect the happiness level of Thai older persons.

Therefore, the objective of this study was to investigate the association between happiness and food-related behaviours and other potential factors such as socio-demographic and economic conditions among older persons in Thailand. The findings of this study should help policymakers and other stakeholders (e.g., public health professionals, social workers, etc.) to better understand what contributes to happy aging and, in particular, how food-related factors can support and foster psychological well-being so that older Thais can continue to make a productive contribution to their family and society. The findings can be used to inform a design in active ageing policies which will enhance health and participation among ageing populations while ensuring adequate security of older persons, taking into account the relationship between food-related behaviours, economic conditions, and happiness.

## Methods

### Study design and population

This study was conducted as part of a nationally-representative sample survey on FV consumption in the Thai population [18]. Data were collected by face-to-face interviews with persons age 60 years or older from the four geographic regions (Central, North, Northeast, and South) and Bangkok during June-December 2021. The multistage stratified sampling design was implemented by NSO. Details of the sampling method are described elsewhere [18].

For the purpose of this study, data from participants age 60 years or older were used for analysis. A total of 1,197 older persons agreed to be interviewed by trained staff using a structured questionnaire. The survey data includes information about socio-demographic characteristics, health status, household chores, home gardening, financial situation, family meals, and food security. Prior to the interview, the researcher obtained the participant’s informed consent. The protocol for the study was approved by the IPSR-Institutional Review Board at Mahidol University.

The study constructed the survey questionnaire through expert consultations on information required for the survey, literature review on validated questions, and development of the final questionnaire (see a supplementary file). The interviews of the participants were carried out by a trained research team using Qualtrics offline survey application. Prior to the survey, heads of villages in each study area were contacted to get a formal approval for data collection. Village heads or coordinators then helped the research team approach the sampled households.

### Measures

#### Dependent variable

Happiness was the main outcome for this study which was measured using a single question: ‘*Presently, what is your level of happiness?’* [19] This single happiness indicator has been used in cross-cultural studies of happiness in many countries around the world [20, 21]. The question includes an 11-point response scale in which the respondent indicates their level of happiness from a number, with potential range from 0 (extremely unhappy) to 10 (extremely happy). The respondents were asked to pick a number that indicates how happy they were at the time of the interview.

#### Independent variables

The independent variables of the study were selected based on the policy framework for active ageing by WHO [9]. The policy framework addresses the three pillars of active ageing: health, participation and security. For the health pillar it is important to improve health status of older persons by preventing and controlling disabilities, chronic disease and premature mortality. For the participation pillar older persons should be encouraged for active participation such as employment activities for formal and informal work, and family community life (such as household chores and home gardening). For the security pillar the framework focuses on social, financial and physical security and needs of older persons which can include having proper meals and food security.

#### Socio-demographic characteristics

Socio-demographic variables include age, sex, marital status, educational attainment, place of residence, and employment. Response was coded as follows:

Age (years) was grouped into 60–64, 65–69, 70–74, and 75 or over. Sex was coded as male or female. Marital status was classified into single, married, and widowed/divorced/separated. Educational attainment was divided into four levels: Below primary, primary, secondary, and post-secondary school. Place of residence was coded as urban or rural area.

#### Active ageing

Active ageing variables were categorised based on three basic pillars of a policy framework for active ageing suggested by WHO [9], which are health, participation, and security.

Health pillar:

Participants were asked to self-assess their own health status. Response was classified into three levels: ‘*Poor, moderate*, and *good/very good*.’

Participation pillar:

##### Employment

Paid employment was coded as ‘*no*’ (unpaid employment) and ‘*yes*’ (paid employment).

##### Household chores

Participants were asked whether they are responsible for any household chores. Each response option was coded as ‘*no*’ or ‘*yes*.’

##### Home gardening

Participants were asked whether they do home gardening. Response was coded as ‘*no’* or ‘*yes*.’

Security pillar:

*Satisfaction with financial situation*: Participants were asked ‘*How satisfied are you with your financial situation?’* They were provided with the following response options: ‘*Not at all satisfied*, *less satisfied*, and *satisfied/most satisfied*.’

##### Family meals

Participants were asked what meal(s) they eat most often with their family/household member(s). Data was coded as not ever eating together, sharing at least one meal a day, and eating every meal together.

##### Food insecurity

This variable is derived from the eight questions of the Food Insecurity Experience Scale (FIES) which was developed by the UN Food and Agriculture Organization [22]. The FIES is a global, standard tool that has been applied worldwide for household or individual food security assessment. The FIES is available in several languages, including Thai. The Thai version of the FIES was calibrated and tested for reliability before data collection [18].

Participants were asked to answer the FIES questions below, with response being either ‘*yes*’ or ‘*no*.’ A ‘*yes*’ response was scored one point. If a respondent answered ‘no’ to any of the FIES questions, the interview then immediately skips to the next section of the questionnaire. The FIES tool is as follows:

*‘Now I would like to ask you some questions about food. During the last 12 months, was there a time when*:

1. *You were worried you would not have enough food to eat?*
2. *You were unable to eat healthy and nutritious food?*
3. *You ate only a few kinds of foods?*
4. *You had to skip a meal?*
5. *You ate less than you thought you should?*
6. *Your household ran out of food?*
7. *You were hungry but did not eat?*
8. *You went without eating for a whole day?’*

The total FIES scores were calculated by summing scores from the eight questions, ranging from zero to eight. The higher score is the more severe the food insecurity. This study classified the food insecurity scores into three groups: 0 (food security), 1–2 (moderate insecurity) and 4–8 (severe insecurity). This score classification is based on previous literature using FIES [23–27].

### Statistical analysis

This study used descriptive statistics (i.e., frequencies and percentages) to present the happiness score, socio-demographic characteristics, food-related behaviours, and other factors. The mean and standard deviation (SD) of happiness were calculated. Then, multivariate regression analysis was used to investigate the statistical association between happiness and other variables, adjusting for the covariates sequentially.

Four, progressively complex models were developed during the analysis. Model 1 examined the relationship between happiness and sociodemographic factors. Model 2 built on Model 1 by adding self-rated health status. Model 3 added employment status, household chores, and home gardening. Model 4 added satisfaction with financial situation, family meals, and food insecurity. Any observed relationship with a p value of 0.05 or less (2-tailed) was considered statistically significant. The multivariate logistic analysis was examined using the SPSS Software Version 22.

## Results

### Characteristics of the participants

Table 1 shows the sociodemographic and other characteristics of the participants. Of the total sample of 1,197 older persons, 70% were age 60–69 years, 55% were female, 68% were married, and 66% had complete primary education. Over half the sample lived in a rural area (58%) and performed unpaid work (58%). Nearly two-thirds of the participants assessed their health status as ‘*moderate*’ or ‘*poor*’ (64%). Almost 70% reported being responsible for household chores, and 24% gardened at home, which 16.1% and 30% of rural and urban residents did home gardening, respectively. More than two-thirds (70%) felt less satisfied or dissatisfied with their current financial situation. The majority (85%) of the sample did not eat every meal with a family/household member(s). Almost 30% had food insecurity.

**Table 1:** **Characteristics of the Sample by Happiness Score** (N = 1,197)

Characteristics	N	%	Happiness score	
			Mean	SD
Age group (years)				
60–64	389	32.5	7.5	1.7
65–69	327	27.3	7.4	1.8
70–74	260	21.7	7.8	1.6
75 or over	222	18.5	7.8	1.7
Sex				
Male	535	44.7	7.6	1.7
Female	662	55.3	7.6	1.7
Marital status				
Single (never-married)	40	3.3	7.4	1.6
Married	812	67.9	7.6	1.7
Widowed/divorced/separated	345	28.8	7.5	1.8
Educational attainment (school)				
Below primary	207	17.2	7.5	1.8
Primary	789	65.9	7.5	1.7
Secondary	144	12.1	7.9	1.5
Post-secondary	58	4.8	8.4	1.2
Place of residence				
Urban	498	41.6	7.7	1.8
Rural	699	58.4	7.6	1.7
Self-rated health status				
Poor	291	24.3	7.1	1.9
Moderate	471	39.4	7.5	1.6
Good/very good	435	36.3	8.0	1.5
Paid employment				
No	688	57.5	7.7	1.8
Yes	509	42.5	7.5	1.6
Household chores				
No	383	32.0	7.7	1.7
Yes	814	68.0	7.5	1.7
Home gardening				
No	908	75.9	7.6	1.8
Yes	289	24.1	7.8	1.5
Satisfaction with financial situation				
Not at all satisfied	285	23.8	6.6	1.8
Less satisfied	559	46.7	7.7	1.5
More/most satisfied	354	29.5	8.2	1.5
Having a daily meal together with family/household member(s)				
Never	489	40.8	7.6	1.8
At least one meal a day	536	44.7	7.5	1.7
Every meal	173	14.4	8.0	1.6
Food insecurity (scores)				
Food secure (0)	850	71.0	7.8	1.6
Moderate insecurity (1–3)	237	19.8	7.3	1.6
Severe insecurity (4–8)	111	9.3	6.8	2.0
Total	1197	100.0	7.6	1.7

### Happiness level

The average happiness score among the sample of older persons was 7.6 (SD = 1.7) (Table 1). Participants age 70 years or over had the highest happiness score at 7.8. Females and males had a similar happiness score (7.6). Married participants (7.6) had higher happiness than participants of other marital status. Participants with post-secondary education had the highest happiness score (8.4) for all variable categories. Participants who lived in an urban area (7.7) had slightly higher happiness than their rural counterparts (7.6). Participants who felt they were in good/very good health were happier than those who felt less healthy. Older persons who performed unpaid work (i.e., unemployed) (7.7) were happier than persons in gainful employment (7.5).

For other characteristics, participants who were not responsible for household chores (7.7) but tended kitchen gardens (7.8) had higher happiness scores compared to those with chores and did not have a kitchen garden. Participants who were more/most satisfied with their financial situation had one of the highest happiness scores at 8.3. Participants who spent time eating every meal with their family/household member(s) had the highest happiness score at 8.0 compared to those who did not share their mealtimes. Participants who were food-secure (7.8) had a higher happiness score than those with moderate or severe food insecurity (7.3 and 6.8, respectively).

### Regression of happiness on sociodemographic factors, food behaviour and other factors

This study tested the data to see if happiness of the participants differed by food behaviours and other factors. The results of the regression analysis (Tables 2 and 3) provide evidence of statistically-significant associations between happiness of older persons, and food behaviours and other factors.

Table 2 shows results of the unadjusted happiness linear regression models. Age, educational attainment, employment status, health status, home gardening, financial satisfaction, family meals, and food insecurity were associated with happiness in older persons. The probability of being happier among persons age 70 years or over and completed secondary school or higher was much higher than those with lower age and educational attainment. There were significant associations of happiness scores with self-rated health status as better than ‘*poor*,’ tending a kitchen garden, having at least some satisfaction with their financial situation, and eating every meal with family/household member(s). Participants with paid work were likely to have 0.2 lower happiness score than people with unpaid work. Participants with food insecurity (at moderate or severe levels) were likely to have 0.5 and 1.0 lower happiness scores than people with food security, respectively.

According to previous research, there is a place of residence difference in the prevalence of home gardening [28]. Therefore, this study conducted the correlation test between home gardening and place of residence. The result shows that the correlations between home gardening (yes) and rural residence was 0.162 (p = 0.000).

**Table 2:** Unadjusted Happiness Linear Regression Models

Variables	B	Std. Error	Beta	p
Age (years) (Reference group: 60–64)				
65–69	− 0.080	0.128	− 0.021	0.531
70–74	0.321	0.136	0.078	0.019
75 or over	0.292	0.143	0.066	0.042
Sex (Reference group: male)				
Female	0.011	0.099	0.003	0.912
Marital status (Reference group: single)				
Married	0.196	0.277	0.053	0.480
Widowed/divorced/separated	0.082	0.286	0.022	0.774
Educational attainment (Reference group: below primary)				
Primary	− 0.003	0.133	− 0.001	0.982
Secondary	0.369	0.184	0.070	0.045
Post- secondary	0.846	0.252	0.106	0.001
Place of residence (Reference group: urban area				
Rural	− 0.108	0.100	− 0.031	0.281
Self-rated health (Reference group: poor)				
Moderate	0.463	0.124	0.132	< 0.001
Good/very good	0.971	0.126	0.274	< 0.001
Employment (Reference group: no paid work)				
Paid work	− 0.232	0.100	− 0.067	0.020
Household chores (Reference group: no)				
Yes	− 0.190	0.106	− 0.052	0.072
Home gardening (Reference group: no)				
Yes	0.234	0.115	0.059	0.042
Financial satisfaction (Reference group: no)				
Less	1.043	0.117	0.305	< 0.001
More/most satisfied	1.606	0.128	0.429	< 0.001
Having meals together with family (Never)				
Some meals	− 0.053	0.107	− 0.016	0.617
Every meal	0.391	0.151	0.081	0.010
Food insecurity (score) (Reference group: food secure (0))				
Moderate (1–3)	− 0.503	0.123	− 0.117	< 0.001
Severe (4–8)	− 0.990	0.170	− 0.168	< 0.001

p – p value; B – Unstandardized coefficient; Beta – Standardized beta coefficient

After adjusting the models (Table 3), some sociodemographic characteristics in Model 1 were found to be related to happiness. Results indicate that the happiness score in older persons was higher for people age 70 years or over, and people with at least secondary school education. People age 70–74 years and 75 years or over were more likely to have 0.4 (B = 0.380; p 0.005 and B = 0.423; p ≤ 0.004, respectively) greater happiness scores than people under age 70 years. People who had secondary school education or higher had 0.5 (B = 0.489; p 0.010) and 1.0 (B = 0.956; p < 0.001) greater happiness scores than people who had education lower than primary school level, respectively.

In Model 2 adding self-rated health status was found to produce a significant association with happiness. Participants who reported living with moderate or good/very good health were likely to have 0.5 (B = 0.460; p < 0.001) and 1.0 (B = 1.040; p < 0.001) higher happiness scores than participants with poor health, respectively.

In Model 3, employment status, household chores, and home gardening were associated with happiness. The inclusion of paid employment and responsibility for household chores decreased the possibility of being happy (B = -0.238; p 0.027 and B = -0.247; p 0.023, respectively). People who kitchen gardened had a 0.3 (B = 0.262; p 0.022) higher happiness score than people who did not garden at home.

In Model 4, the inclusion of satisfaction with financial situation, family meals and food insecurity decreased the regression coefficients of previous variables. Statistically significant associations of financial situation, family meals, and food insecurity were found with happiness. People who were less or more/most satisfied with their financial situation had 0.9 (B = 0.846; p < 0.001) and 1.3 (B = 1.293; p < 0.001) higher happiness score than people who were dissatisfied with their financial situation, respectively. The highest probability of being happy was found in people who ate every meal with family member(s) (B = 0.344; p 0.018). The coefficient of food insecurity was 0.4 (B = -0.437; p 0.008). This implies that people who had severe food insecurity had 0.4 lower happiness score than people with food security. The adjusted R square in this model also increase substantially from 0.085 to 0.175. Thus, financial situation, family meals and food insecurity can be considered significant predictors of happiness in Thai older persons.

**Table 3:** Multiple Regression of Happiness by Socio-demographic Characteristics and Food and Health-related Behaviours

Variables	Model 1				Model 2				Model 3				Model 4			
	B	Std. Error	Beta	p	B	Std. Error	Beta	p	B	Std. Error	Beta	p	B	Std. Error	Beta	p
Age (years) (Reference group:60–64)																
65–69	− 0.043	0.127	− 0.011	0.736	− 0.001	0.124	0.000	0.993	− 0.030	0.124	− 0.008	0.808	− 0.054	0.119	− 0.014	0.650
70–74	0.380	0.137	0.092	0.005	0.467	0.133	0.113	< 0.001	0.384	0.135	0.093	0.005	0.212	0.130	0.051	0.104
75 or over	0.423	0.148	0.096	0.004	0.571	0.145	0.130	< 0.001	0.427	0.152	0.097	0.005	0.323	0.146	0.074	0.027
Sex (Reference group: male)																
Female	0.150	0.107	0.044	0.161	0.244	0.105	0.071	0.020	0.262	0.109	0.076	0.017	0.181	0.105	0.053	0.084
Marital status (Reference group: single)																
Married	0.546	0.283	0.149	0.054	0.517	0.275	0.141	0.061	0.510	0.274	0.139	0.063	0.418	0.264	0.114	0.113
Widowed/divorced/separated	0.337	0.288	0.089	0.242	0.318	0.280	0.084	0.257	0.300	0.279	0.080	0.283	0.327	0.266	0.087	0.219
Education attainment (school) (Reference group: below primary)																
Primary	0.055	0.133	0.015	0.681	− 0.039	0.130	− 0.011	0.763	0.032	0.131	0.009	0.809	0.081	0.126	0.022	0.520
Secondary	0.489	0.190	0.093	0.010	0.374	0.185	0.071	0.044	0.406	0.185	0.077	0.029	0.324	0.177	0.062	0.068
Post-secondary	0.956	0.257	0.120	< 0.001	0.815	0.251	0.102	0.001	0.803	0.253	0.101	0.002	0.458	0.244	0.058	0.061
Place of residence (Reference group: urban area)																
Rural	− 0.055	0.100	− 0.016	0.585	− 0.077	0.097	− 0.022	0.430	− 0.094	0.098	− 0.027	0.339	− 0.038	0.093	− 0.011	0.687
Health:																
Self-rated health (Reference group: poor)																
Moderate					0.460	0.123	0.132	< 0.001	0.505	0.123	0.144	< 0.001	0.353	0.119	0.101	0.003
Good/very good					1.040	0.127	0.293	< 0.001	1.100	0.129	0.310	< 0.001	0.883	0.124	0.249	< 0.001
Participation:																
Paid employment (Reference group: no)																
Yes									− 0.238	0.108	− 0.069	0.027	− 0.190	0.103	− 0.055	0.065
Household chores (Reference group: no)																
Yes									− 0.247	0.109	− 0.067	0.023	− 0.208	0.104	− 0.057	0.045
Home gardening (Reference group: no)																
Yes									0.262	0.114	0.066	0.022	0.244	0.110	0.061	0.027
Security:																
Satisfaction with financial situation (Reference group: not at all satisfied)																
Less satisfied													0.846	0.119	0.247	< 0.001
More/most satisfied													1.293	0.134	0.345	< 0.001
Having meals with family member(s) (Reference group: never)																
At least one meal a day													− 0.079	0.102	− 0.023	0.441
Every meal													0.344	0.145	0.071	0.018
Food insecurity (score) (Reference group: secure (0))																
Moderate insecurity (1–3)													− 0.117	0.120	− 0.027	0.328
Severe insecurity (4–8)													− 0.437	0.164	− 0.074	0.008
**(Constant)**	**6.796**	**0.337**		**0.000**	**6.249**	**0.337**		**0.000**	**6.428**	**0.343**		**0.000**	**5.920**	**0.341**		**0.000**
**Adjusted R Square**	**0.024**				**0.076**				**0.085**				**0.175**			

p – p value

B – Unstandardized coefficient

Beta – Standardized beta coefficient

## Discussion

This study examined data on the association between happiness and financial satisfaction, food insecurity, and sharing family meals among Thai older persons. This study offers new insights for future public policy and interventions for engaging, inspiring, and enabling people to be healthy and happy in later life by enhancing security of older persons through reducing financial hardship, sharing family mealtimes, and reducing food insecurity. If older persons have support for improved diets in older age through provision of security and care against economic hardships and limited access to nutritious food, then scientific evidence of extra psychological gains from a healthy diet might enable older persons and their families to make heathier foods choices and, thus, improve the health of the population in the long-run.

The multivariate analysis of influential factors in the present study shows that Thai older persons can be affected by happiness in different ways depending on their sociodemographic characteristics, and the three pillars of active ageing measures, namely, health, participation, and security. The study documented a strong association between security and family care factors and happiness, after controlling for various socio-demographic factors and elements of health and participation. Particularly, education and employment were found to be significantly associated with happiness in the Models #1–3. However, these variables became insignificant after adding variables on satisfaction with financial situation, family meals and food insecurity to the model. This points out that the major predictors of increased happiness were at a certain level of financial satisfaction, opportunity of eating every meal with family/household member(s), and the lack of experiencing severe food insecurity.

The older person’s economic environment has a particularly significant effect on active ageing [9]. The present study found a strong association between perceived financial satisfaction and happiness in older persons. Low financial satisfaction can be attributed to financial worries or hardship which can erode mental health [29]. This suggests that financial satisfaction may be one of the most important socio-economic determinants of happiness, and can be used as a subjective indicator to monitor and prevent hardship and, thus, promote health and wellbeing of older persons in the long-run.

Sharing the family meal is an important component of happy aging. This study found that older persons who had every meal with family were likely to be happier than those who did not eat meals with family/household member(s). Mealtimes can yield benefits that go beyond food. Not only can it improve nutritional status and reduce health risk behaviours [30, 31], sharing meals is linked to psychological wellbeing. Previous studies showed that being included in family mealtimes was associated with wellbeing of adults, older persons, and parents across such dimensions as self-esteem, depressive symptoms, and stress [30, 32, 33]. This is because sharing the family meal provides household members a chance to discuss issues that may be bothering them, reaffirm family values, and improve or strengthen the family bond. Those attributes are contributors to greater happiness. Findings from the present study point to the importance of sharing the family meal and, in particular, the potential for enhancing psychosocial wellbeing of older persons and their family.

Severe food insecurity was defined as a major barrier to happiness of Thai older persons in this study. It has also been pointed out that older persons who experienced the same severe level of food insecurity were less likely to be happy, after controlling for shared mealtimes with family. That finding is consistent with several other studies which found indirect and long-term consequences of food insecurity and its adverse effect on health and wellbeing. A global study using the FIES tool found that experience of food insecurity contributed to both poor physical health and lower subjective well-being of individuals [34]. The negative effect of food insecurity was even stronger in higher-income countries [35]. Similarly, a negative association between hunger (a likely indicator of severe food insecurity) and subjective well-being was observed, controlling for individual income and food security status [36]. In urban Ethiopia, a macroeconomic event, e.g., food price shocks, adversely affected subjective well-being [37]. The findings suggest that externalities could play a significant role in the relationship between food insecurity and subjective well-being. Reducing food insecurity will not only increase the subjective well-being of those being lifted out of hunger, but could also improve the well-being of the people surrounding them. Therefore, food insecurity can be used as a social development indicator that represents security in the life of the population.

Improving food security at the individual level will directly support a country’s progress toward achieving Sustainable Development Goal (SDG) #2 (Hunger), and will contribute to SDG #3 (Health and Well-Being) that includes subjective well-being or happiness as one of its indirect indicators. According to the Sustainable Development Report 2022, Thailand’s performance on the subjective wellbeing indicator declined [38]. The subjective well-being score was only slightly higher than midpoint of the potential range (5.6 out of 10). Therefore, the role of food security for health and well-being, and its implications for the economic performance of older persons requires special attention from policy makers and other stakeholders.

The findings of this study stress the importance of the Thai older person’s financial situation, ability to share in family meals, and food insecurity compared to other variables in the study. Education and employment were found to be significantly associated with happiness in regression Models #1–3. However, these factors became insignificant after adding variables on satisfaction with financial situation, sharing family meals, and food insecurity. This suggests that a favourable financial situation, sharing family meals, and food security are important contributors to active ageing, and that they can produce significant health and well-being benefits for happiness of Thai older persons.

This study has some limitations. First, this research used a cross-sectional design, and that precludes the ability to assert cause and effect relationships. A longitudinal or experimental study may be needed to examine cause-effect relationships between active ageing variables and happiness. Second, a subgroup analysis for examining influence of factors related to happiness on different demographic groups such as sex or age was not conducted in this study and would be important area for further analysis. Testing for interactions can also be performed to determine whether influence of factors differ between different subgroups. Third, although the findings can be generalised to the entire older Thai population, the selection of the sample targeted general older persons who had the ability to participate in the study; those with disabilities were not targeted. Since an active ageing approach aims to “*extend healthy life expectancy and quality of life for all people as they age, including those who are frail, disabled, and in need of care* [9],” additional research including this vulnerable group is needed. Fourth, the survey was taken during COVID-19 pandemic in Thailand that can make Thai older persons more prone to health and nutrition risks especially people in low-income groups [39, 40], which can be negatively associated with happiness. Therefore, these circumstances may impact or influence the application or interpretation of the study results. Fifth, although the variables in this study covered all three basic pillars of active ageing (security, health, and participation), other factors (e.g., policies and actions addressing the three basic pillars) were not investigated. Therefore, future research should explore the effect of environmental factors on active ageing.

## Conclusions

Consistent and comprehensive information on food-related behaviours and happiness in an ageing population has so far been lacking in many countries in Southeast Asia, especially Thailand. This study used multivariate analysis to identify statistically-significant determinants of happiness in Thai older persons. The top three determinants associated with a higher likelihood of being happy are financial satisfaction, sharing the family meal, and food insecurity. The findings suggest the need for a shift in policy from a focus on short-term strategies (i.e., food and nutrition interventions) to longer-term efforts that ensure financial self-sufficiency and food security for older persons and their households. It is essential for policy makers to recognize the heterogeneity of the determinants of happiness across various dimensions of the lives of older persons, particularly those related to pillars of the active ageing process.

### Electronic supplementary material

Below is the link to the electronic supplementary material.

Supplementary Material 1

## Data Availability

Data available on request to the corresponding author due to privacy for research participants.

## List of abbreviations

FIES: Food Insecurity Experience Scale
FV: Fruit and vegetable
NSO: National Statistical Office
SD: Standard deviation
WHO: World Health Organization
