# Supplementary material for: Financial satisfaction, food security, and shared meals are foundations of happiness among older persons in Thailand
Source: BMC Geriatr. 2023 Oct 24;23:690. doi: 10.1186/s12877-023-04411-1 (PMC10599056; doi:10.1186/s12877-023-04411-1)
Supplement: Supplementary file 1 — Supplementary Material 1 [file 12877_2023_4411_MOESM1_ESM.docx]

**A Longitudinal Study on Fruit and Vegetable Eating Behaviors Questionnaire (in 2021)**

Q1. Age

……… years old

Q2. Gender

[ ] 1. Male [ ] 2. Female

Q3. Marital status

[ ] 1. Single [ ] 2. Married

[ ] 3. Widowed, divorced or separated

Q4. Education attainment (school)

[ ] 1. Below primary [ ] 2. Primary

[ ] 3. Secondary [ ] 4. Post-secondary

Q5. Place of residence

[ ] 1. Urban [ ] 2. Rural

Q6. Self-rated health status

[ ] 1. Poor [ ] 2. Moderate

[ ] 3. Good [ ] 4. Very food

Q7. Paid employment

[ ] 1. No [ ] 2. Yes

Q8. Are you responsible for any household chores?

[ ] 1. No [ ] 2. Yes

Q9. Do you do garden at home?

[ ] 1. No [ ] 2. Yes

Q10. How satisfied are you with your financial situation?

[ ] 1. Not at all satisfied [ ] 2. Less satisfied

[ ] 3. More satisfied [ ] 4. Most satisfied

Q11. what meal(s) you eat most often with their family/household member(s)?

[ ] 1. Never [ ] 2. At least one meal a day

[ ] 3. Every meal

Q12. Now I would like to ask you some questions about food. During the last 12 months, was there a time when:

| **Questions** | **No (0)** | **Yes (1)** |
| --- | --- | --- |
| 1. You were worried you would not have enough food to eat? |  |  |
| 2. You were unable to eat healthy and nutritious food? |  |  |
| 3. You ate only a few kinds of foods? |  |  |
| 4. You had to skip a meal? |  |  |
| 5. You ate less than you thought you should? |  |  |
| 6. Your household ran out of food? |  |  |
| 7. You were hungry but did not eat? |  |  |
| 8. You went without eating for a whole day?’ |  |  |

Q13. What level of happiness are you currently at present (based on a score of 0-10, 0 means not happy at all and 10 means happiest)?

0 1 2 3 4 5 6 7 8 9 10
